# The effects of *Rhodopseudomonas palustris* on the improvement of agronomic traits and key enzyme-coding genes related to polysaccharide biosynthesis in *Codonopsis pilosula*

**DOI:** 10.1371/journal.pone.0319989

**Published:** 2025-06-03

**Authors:** Wanhua Wang, Shuhui Gao, Yi Sun, Hong Yang, Jinlong Li, Jing Li, Xianhui Zheng, Guane Yang

**Affiliations:** 1 School of Pharmacy, Shanxi Medical University, Taiyuan, Shanxi, China; 2 Medicinal Basic Research Innovation Center of Chronic Kidney Disease, Ministry of Education, Shanxi Medical University, Taiyuan, China; 3 Shanxi Provincial Key Laboratory of Drug Synthesis and Novel Pharmaceutical Preparation Technology, Shanxi Medical University, Taiyuan, China; ICAR - National Rice Research Institute, INDIA

## Abstract

The content of *Codonopsis pilosula* polysaccharide (CPPS) is a critical indicator of the quality and quantity of *Codonopsis pilosula (C. pilosula)*, though the biosynthetic mechanism of CPPS accumulation through the application of *Rhodopseudomonas palustris (R. palustris)* remains unclear. Therefore, when planting *C. pilosula*, we applied *R. palustris* through spraying and root irrigation (10 ml), and harvested its fresh roots, stems and leaves as experimental materials. Agronomic traits and CPPS content were determined, while transcriptome sequencing was analyzed, with gene expression verified by fluorescence quantitative PCR. The results revealed that the phenotype of *C. pilosula* was improved, and the content of CPPS in roots, stems, and leaves increased by 90.22%, 61.11%, and 20.00%, respectively. Following sequencing, 10,880, 8,578, and 12,340 differentially expressed genes (DEGs) were identified in response to *R. palustris* application. The DEGs in each tissue were primarily enriched in starch and sucrose metabolism, phenylpropanoid and flavonoid biosynthesis, glycolysis/gluconeogenesis, MAPK signaling pathways, and plant hormone signal transduction. A total of 12 genes encoding sucrose synthase (SUS), hexokinase (HK), β-fructofuranosidase (sacA), and fructokinase (scrK) were significantly upregulated in the tissues, with expression levels higher in roots than in stems and leaves. Additionally, 10 genes encoding proteins with jasmonate ZIM domains (JAZ), coronatine-insensitive protein 1 (COI1), and transcription factor MYC2 (MYC2) may be closely associated with the improvement of agronomic traits in *C. pilosula*. This study demonstrated that *C. pilosula’s* response to exogenous *R. palustris* induced the activation of SUS, HK, sacA, scrK, JAZ, COI1, and MYC2 activities. The upregulation of genes regulating these enzymes contributed to the increased CPPS content and the enhancement of agronomic traits in *C. pilosula*. These findings provide a reference for cultivating high-quality *C. pilosula* at the molecular level.

## Introduction

*C. pilosula* is considered one of the traditional Chinese medicinal materials, highly valued for its medicinal and edible qualities. Its demand in traditional Chinese medicine has continuously increased [1]. Medicinal plants have been used for therapeutic, religious, cosmetic, nutritional, and beautification purposes since ancient times and humanity of all civilizations and culture are familiar with their usage [2,3]. CPPS, one of the primary pharmacodynamic components of *C. pilosula*, have biological functions such as enhancing human immunity, anti-tumor effects, participation in blood sugar regulation, and delaying ageing [4–6]. Therefore, CPPS content is an important indicator of *C. pilosula* quality [7,8]. As demand for artificially cultivated *C. pilosula* continues to rise, the reliance on chemical fertilizers in traditional cultivation methods has become a significant issue, not only causing environmental pollution but also affecting the yield and quality of *C. pilosula* and further affect human life and health. Previous research results have indicated that a strain with potential to promote plant growth was isolated from the rhizosphere of *C. pilosula*. This strain was identified as *Bacillus licheniformis* YB06, which exhibits a positive effect on enhancing the germination rate of *C. pilosula* seeds and promoting seedling growth [9]. Tingting Jin et al. [10] found that *Klebsiella michiganensis* can facilitate the growth of *C. pilosula* seedlings and increase the activities of sucrose and urease enzymes in the rhizosphere soil of the seedlings. However, we have not encountered any relevant studies on the impact of *R. palustris* on the growth and quality of cultivated *C. pilosula*. In recent years, microbial inoculants have attracted considerable attention due to their ability to significantly reduce the use of chemical fertilizers [11]. *R. palustris*, one of the microbial agents [12,13], can significantly promote crop growth, improve yield and quality, reduce heavy metal pollution, and aid in sewage treatment [14–17]. For instance, the results obtained by Jiayu Chang et al. demonstrated that *R. palustris* can significantly improve the biomass and quality of *Astragalus membranaceus,* Compared to the control group, the group treated with *R. palustris* biofertilizer showed a 43.2% increase in the content of calycosin-7-O-β-D-glucoside in the roots of *Astragalus membranaceus* [18]. Xuemin Wei et al. conducted research on the abiotic stress of cadmium (Cd) in *Salvia miltiorrhiza* and found that *R. palustris* could increase the accumulation of total tanshinones by 40.45% [19]. Our research team has also initially explored the application of *photosynthetic bacteria* in the cultivation of traditional Chinese medicinal materials such as *C. pilosula*. The research results indicated that *R. palustris* could promote the accumulation of syringin (by 11.22%) and CPPS (by 15.91%) in *C. pilosula*, while also increasing root length by 13.55% [20]. Nevertheless, *R. palustris*, with its environment-friendly and ecologically safe characteristics, offers considerable potential in environmental protection [21,22]. For instance, Su et al. found that the application of *R. palustris* significantly reduced Cd contamination in paddy soil while improving rice growth and yield [23]. Similarly, Khuong NQ et al. demonstrated that microbial fertilizers containing *R. palustris* can significantly increase the concentrations of available nitrogen and soluble phosphorus in soil, thereby promoting nutrient absorption and enhancing sesame yield [24]. Given the importance of *R. palustris* in enhancing crop growth and quality, as well as its potential in traditional Chinese medicinal materials, it is crucial to investigate its effects on improving the agronomic traits of *C. pilosula* and the regulatory mechanisms influencing the accumulation of CPPS.

CPPS biosynthesis is a complex process that involves three key metabolic pathways: starch and sucrose metabolism, amino sugar and nucleotide metabolism, and glycolysis and gluconeogenesis metabolism. These pathways work together to promote the accumulation of CPPS [25]. Starch and sucrose metabolism play a predominant role in this process, primarily by converting glucose-6-phosphate (Glu-6-P) and fructose-6-phosphate (Fru-6-P) into uridine diphosphate glucose (UDP-Glu) and guanosine diphosphate mannose (GDP-Man). Li W. et al. verified the crucial role of starch and sucrose metabolic pathways in determining the quality and flavor of peanut seeds through transcriptome sequencing and KEGG analysis of differential genes [26]. Wang S. et al. conducted a transcriptome analysis on bamboo and found that starch and sucrose metabolism were significantly correlated with growth and soluble sugar content [27]. In plants, UGP2 catalyzes the conversion of glucose-1-phosphate (Glu-1-P) into UDP-Glu, an essential precursor for UDP-sugars. Xu Z. et al. discovered, via transcriptome sequencing of cotton, that genes encoding UGP2 play a vital role in fiber development, offering an opportunity for plant researchers to explore the mechanisms underpinning cotton fiber development [28]. In rice, UDP-glucose pyrophosphorylase (UGPase) promotes callose biosynthesis, further illustrating the essential role of UGPase in polysaccharide biosynthesis [29]. Additionally, UGPase catalyzes a reversible reaction that produces UDP-glucose (UDPG), which serves as a precursor for hundreds of glycosyltransferases across all organisms [30]. In *Lactobacillus acidophilus*, the overexpression of UGPase accelerates the growth rate of *Streptomyces carneus* but decreases its freeze-dried survival rate [31]. UDP-Glu subsequently acts as a substrate for further conversion into other UDP-sugars, such as UDP-glucuronic acid and UDP-arabinose. Under the catalytic action of glycosyltransferases, these active monosaccharides contribute to the accumulation of CPPS.

Previous researchers explored the influence of different concentrations of *R. palustris* on the active ingredients and trace elements of *C. pilosula* [20], but they did not analyze the mechanism of the accumulation of the active ingredient CPPS in *C. pilosula* following root irrigation and spraying treatment with *R. palustris*. RNA-seq sequencing technology provides an opportunity to investigate the molecular mechanism responsible for the accumulation of CPPS content. Therefore, this study used three-month-old *C. pilosula* plants as experimental materials to explore the effects of exogenous *R. palustris* on the agronomic traits of *C. pilosula*, the content of CPPS in its roots, stems, and leaves, and the molecular mechanism underlying CPPS accumulation. Transcriptome sequencing analysis was employed to reveal the differentially expressed genes (DEGs) in the roots, stems, and leaves of *C. pilosula* after *R. palustris* treatment. This provides a foundation for understanding the regulatory mechanism of CPPS biosynthesis and accumulation, as well as offering a reference for cultivating high-quality *C. pilosula* at the molecular level.

## Materials and methods

### Cultivation of potted *C. pilosula* and sample harvesting and processing

dx.doi.org/10.17504/protocols.io.x54v9rzy4v3e/v1

The *R. palustris* inoculant was isolated, identified, and preservedand in the Traditional Chinese Medicine Teaching and Research Section of Shanxi Medical University. The strain was activated and purified using a photosynthetic bacterial culture medium containing 1640 mg of sodium acetate, 75 mg of CaCl₂·2H₂O, 200 mg of MgSO₄·7H₂O, 20 mg of EDTA, 1000 mg of yeast extract, 4900 mg of K₂HPO₄, 1320 mg of (NH₄)₂SO₄, 600 mg of KH₂PO₄, 11.8 mg of FeSO₄·7H₂O, 1 mL of trace elements, and deionized water to a total volume of 1000 mL, with a pH of 6.8–7.2. The culture was maintained at 28 ± 2°C, with a light intensity of 1200 Lux, and continuous light for 24 hours. After 3 days of cultivation, the viable cell count reached ≥2.0 × 10⁸ CFU/mL.

The research was conducted at the Plant Culture Room, School of Pharmacy, Shanxi Medical University, Zhongdu Campus, Jinzhong City, Shanxi Province, China. The conditions in the culture room were as follows: daytime temperature 25 ± 2°C, nighttime temperature 15 ± 2°C, light intensity 1500 Lux, 12 hours of light per day, and employed seeds harvested from *C. pilosula* grown in the fields of Pingshun County, Shanxi Province, which were authenticated as *C. pilosula* seeds by Professor Bai Yun’e from Shanxi Medical University. The soil used for planting *C. pilosula* was purchased from Jiuwo Agricultural Technology Co., Ltd., Shandong Province, China. The soil had an organic matter content ≥50% and a neutral pH. Prior to the experiment, the soil was sterilized at 120°C for 30 minutes in an oven. The experiment included two treatment groups: a control group (CK) with distilled water and a treatment group with a 100-fold dilution of *Rhodopseudomonas palustris* biofertilizer (*R. palustris* Z100). The plants were grown in pots measuring 14 cm in height, with a base width of 7.5 cm and a top width of 10.2 cm, filled with 500 g of soil. Thirty pots were used for each treatment, with three replicates per group, totaling 180 pots. After 90 days, the plants were harvested, and 30 complete *C. pilosula* plants were randomly selected from each treatment group for analysis, with 3 replicates per group, each consisting of 10 plants. The roots, stems, and leaves of the plants were collected as experimental materials.

After sampling, one portion of the samples was thoroughly rinsed with distilled water, and the surface water was absorbed using blotting paper. The root length and plant height of *C. pilosula* were measured using a tape measure. The diameter of the main root, main stem branches, leaf length, leaf width, and main stem diameter were measured with a vernier caliper. The number of leaves, lateral roots, and main stem branches were counted manually. The samples were then dried at 45–50°C, ground, and passed through a 60-mesh sieve. The CPPS content of *C. pilosula* was determined using the phenol-sulfuric acid method. The other portion of the samples was cleaned with enzyme-free water, the surface water was absorbed with blotting paper, and the samples were wrapped in aluminum foil. These samples were quickly frozen with liquid nitrogen and stored in a -80°C freezer for transcriptome sequencing of *C. pilosula*.

### Extraction of RNA and library construction

Using roots, stems, and leaves from two treatments, with three biological replicates as materials, each tissue was placed in a mortar and ground in liquid nitrogen. Total RNA was extracted using TRIzol reagent. Agarose gel electrophoresis (gel concentration: 1.2%; 0.5×TBE electrophoresis buffer; 150v for 15 minutes) was performed to assess the integrity of the RNA. The purity of the RNA was detected using a spectrophotometer (ThermoScientific, USA) under OD260/OD280 conditions. The VAHTS Universal V6 RNA-seq Library Prep Kit was used to construct the transcriptome libraries. Sequencing was performed on the Illumina Novaseq6000 sequencing platform, generating 150 bp paired-end reads. The raw data (raw reads) in fastq format were processed using Trimmomatic [32] to remove reads containing poly-N and low-quality reads, resulting in clean reads. The clean reads were then assembled into expressed sequence tags (contigs), and the transcripts were de novo assembled using Trinity [33] software. Based on sequence similarity and length, the longest Unigene was selected, and the obtained Unigenes were annotated separately in functional databases such as the Non-Redundant Protein Database (NR), Clusters of Orthologous Groups (KOG), Gene Ontology (GO), Swiss-Prot Protein Database (Swiss-Prot), evolutionary genealogy of genes: Non-supervised Orthologous Groups (eggNOG), Kyoto Encyclopedia of Genes and Genomes (KEGG), and Protein Family Database (Pfam).

### Transcriptome data analysis methods

The number of reads aligned to Unigenes in each sample was obtained using the software bowtie2 [34], and using eXpress [35] software to calculate the FPKM [36] values of Unigenes. DESeq2 [37] software was used to calculate the differential multiples and the negative binomial distribution (NB) test was used to determine the significance of the differences. By default, DEGs were screened based on q < 0.05 and fold change ≥ 2, which was considered statistically significant. The spliced Unigenes were used as the database, and the expression abundance of each Unigene in each sample was identified by sequence similarity alignment. Principal component analysis (PCA) was performed using Unigene expression to investigate the distribution of samples. Based on the hypergeometric distribution, R software was used to analyze the GO enrichment and KEGG pathway enrichment of DEGs, respectively. The DEGs involved in the biosynthesis of CPPS were up-regulated after *R. palustris* treatment, and the FPKM values of the key DEGs were analyzed.

### Verification of expression level by qRT-PCR

Each tissue sample was placed into a mortar and ground in liquid nitrogen. Total RNA was extracted using the RNAQUEOUS KIT, Ambion-1912, and stored in a -80°C freezer for later use. A 0.5 microgram portion of RNA was taken for reverse transcription to synthesize the first strand of cDNA, which was then placed in a -80°C freezer after synthesis was completed. The qRT-PCR primers were designed according to each nucleotide sequence fragment using Premier 5.0 software (Table 1). The PCR mix was prepared using the 2×PerfectStartTM Green qPCR SuperMix kit and then subjected to 45 cycles of PCR amplification under the following cycling conditions: 94°C for 30 seconds, followed by 94°C for 5 seconds and 60°C for 30 seconds. The reaction was processed on the Roche LightCycler 480 II for detection. The expression levels were calculated using the 2-∆∆Ct method, and the expression amount was calculated accordingly. In this experiment, glyceraldehyde-3-phosphate dehydrogenase (GAPDH) was used as the internal reference gene to validate the expression levels of key enzyme genes (TRINITY_DN19905_c0_g1_i5_6, TRINITY_DN14015_c0_g1_i3_1, TRINITY_DN22021_c1_g1_i5_1, TRINITY_DN17387_c0_g1_i13_7) from the transcriptome database of *C. pilosula* through quantitative real-time PCR (qRT-PCR) analysis.

**Table 1:** Design of PCR Primer.

Gene Symbol	Forward primer(5->3)	Reverse primer(5->3)	Product length(bp)	Tm(°C)
TRINITY_DN19905_c0_g1_i5_6	CTTAAGCGCATAAAGCAGCAA	TTGTCCACAAGTGGTGCCTA	96	60
TRINITY_DN14015_c0_g1_i3_1	AAGGATTTGCCATTGAGGATAC	GCGCATATCTAGGCCTCTTC	86	60
TRINITY_DN22021_c1_g1_i5_1	GACAGACACGATTGGTATGC	CCTATACCCACGTCCATACT	89	60
TRINITY_DN17387_c0_g1_i13_7	GCCTGGTGCTGATATGTCT	CTCATCGATTTCATGGTGAAGT	82	60
GAPDH	TGCTTCGTTCAACATCATTC	CATAACTGGCTGCCTTCTCC	164	60


$${\text{2}}^{\text{-}\Delta \Delta \text{Ct}}={\text{2}}^{\text{-(}\Delta \text{Ct Experimental group samples}-\Delta \text{Ct control group samples)}}=2^{\text{-}\Delta \text{Ct Experimental group samples}}/2^{\text{-}\Delta \text{Ct control group samples}}$$ (1)

## Results

### The effect of *R. palustris* on the agronomic traits of *C. pilosula*

**Fig 1:**
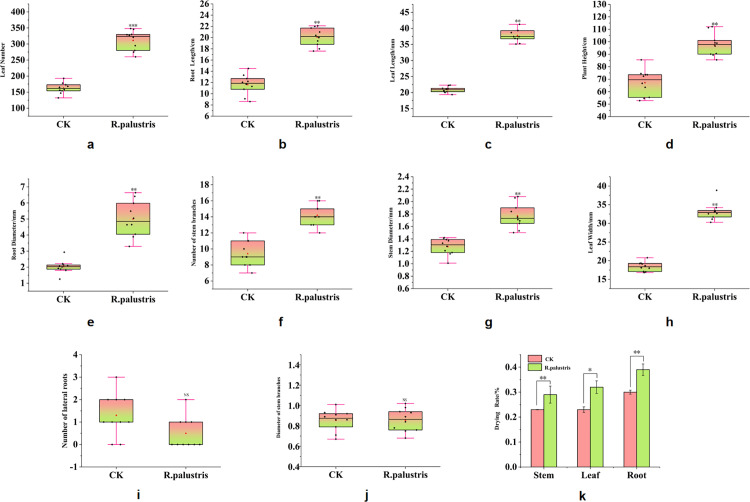
Effects of *Rhodopseudomonas palustris* on the agronomic traits of *Codonopsis pilosula.* (a) Number of leaves; (b) Root Length; (c) Leaf length; (d) Plant height; (e) Root Diameter; (f) Diameter of stem branches; (g) stem Diameter; (h) Leaf width; (i) Number of lateral roots; (j) Number of stem branches; (k) Drying rate. In each box, black dots represent sample size (n = 10); red triangles represent mean values; the centerline represents the median; box limits indicate the 25th and 75th percentiles; whiskers extend 1.5 times the interquartile range from the 25th and 75th percentiles. Error bars represent standard errors (n = 10). In Figs a-k, “*”, “**”, “***”, and “NS” indicate statistically significant differences with p < 0.05, p < 0.01, p < 0.001, and p > 0.05, respectively, according to the independent samples t-test.

Fig 1 shows the effect of a 100-fold dilution of *R. palustris* on the agronomic traits of *C. pilosula*. Compared to the distilled water control group, the root diameter, number of main stem branches, root length, plant height, diameter of the main stem, number of leaves, leaf length, and leaf width of *C. pilosula* exhibited a positive growth effect. However, there was minimal impact on the number of lateral roots and the diameter of the main stem branches (Fig 1i-1j). There was also a notable increase in the drying rate of roots, stems, and leaves of *C. pilosula*. Following *R. palustris* treatment via irrigation and spraying, the number of leaves increased by 90.56% compared to the distilled water control group (Fig 1a). The length of the main root increased by 8.47 cm, which was 72.91% higher than in the distilled water control group (Fig 1b). The leaf length increased by 17.87 mm, or 80.80%, compared to the distilled water control group (Fig 1c). Plant height, root diameter, the number of main stem branches, main stem diameter, and leaf width increased by 44.90%, 1.46%, 51.06%, 38.79%, and 79.93%, respectively (Figs 1d-1h). The drying rates of roots and leaves in *C. pilosula* increased by 11.85% and 14.24%, respectively, though the drying rate of stems increased by only 8.15% (Fig 1k). The results suggest that the application of *R. palustris* can effectively improve the agronomic traits of *C. pilosula*.

### The effect of *R. palustris* on the content of CPPS

**Fig 2:**
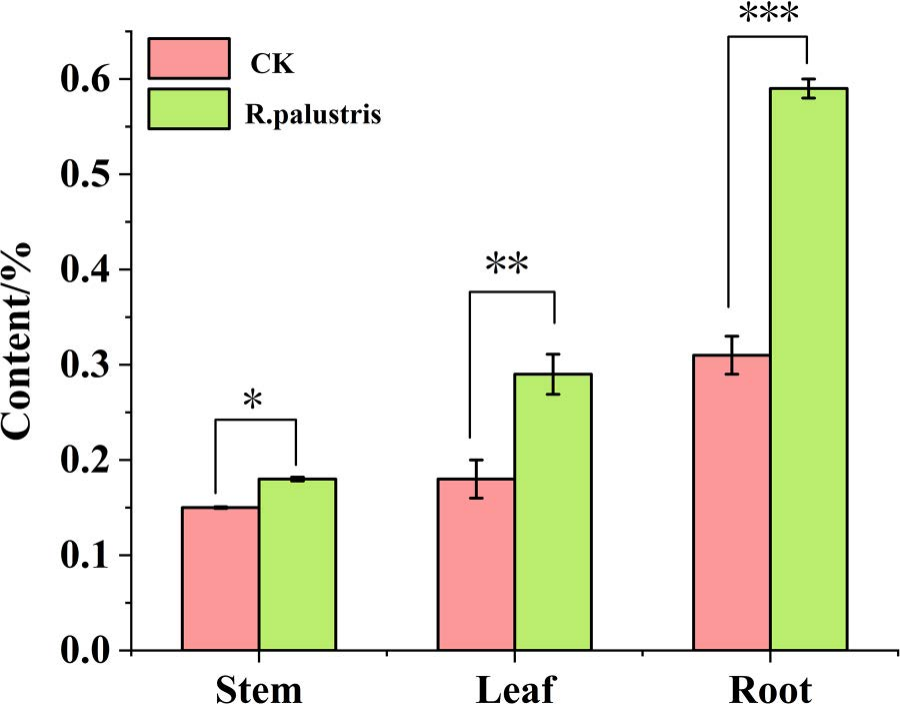
Effect of *Rhodopseudomonas palustris* on the content in roots, stems, and leaves of *Codonopsis pilosula* polysaccharide (n = 10). In Fig 2, “*”, “**”, and “***”, indicate statistically significant differences with p < 0.05, p < 0.01, and p < 0.001 respectively, according to the independent samples t-test.

Fig 2 shows the effect of a 100-fold dilution of *R. palustris* on the content of CPPS. As shown in Fig 2, the highest content of CPPS was found in the roots, reaching 59%, followed by the leaves at 29%, with the stems having the lowest content at 18%. Compared to the control group treated with water, after spraying and root irrigation with *R. palustris*, the polysaccharide content in the roots increased by 90.22%, in the leaves by 61.11%, and in the stems by 20.00%. Overall, the trend was roots> leaves> stems. This indicates that treatment with *R. palustris* can significantly increase the content of CPPS, the active ingredient in *C. pilosula*.

### Analysis of sequencing and database annotation results of *C. pilosula*

**Fig 3:**
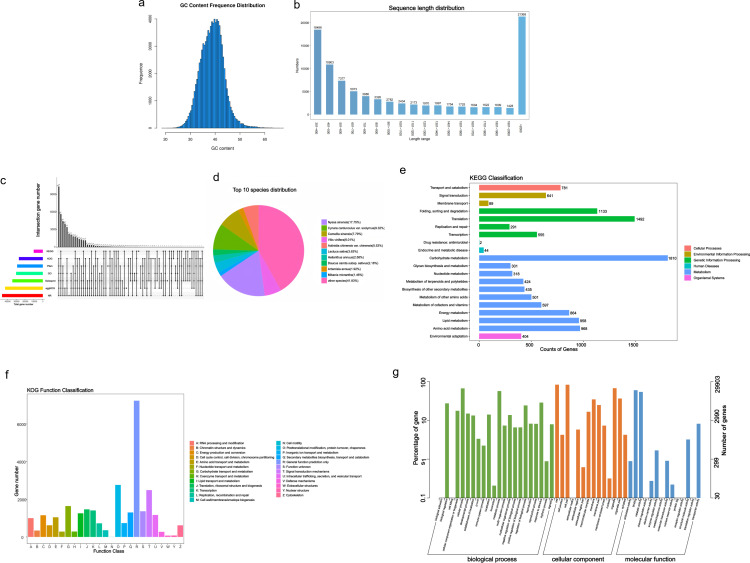
Analysis of sequencing and annotation results of *Codonopsis pilosula.* (a) GC Content Chart; (b) Unigene Length Distribution Chart; (c) Venn Diagram of Annotation Databases; (d) Top 10 Species Distribution Chart in NR Database; (e) KEGG Annotation Chart at Level 2, with the numbers on the right of the bars representing the number of genes annotated to the Level 2 pathway; (f) KOG Functional Classification Chart; (g) GO Functional Classification Chart.

To comprehensively study the effects of *R. palustris* on the roots, stems, and leaves of *C. pilosula*, transcriptome analysis was conducted. A total of 18 samples were sequenced, yielding 121.25 G of clean data. The clean base of each sample ranged from 5.48 to 7.02 G. The Q30 base ratio ranged from 93.2% to 93.88%, and the average GC content ranged from 43.32% to 44.65%, as shown in Fig 3a. A total of 91,627 Unigenes were assembled, with details provided in Table 2. The distribution of Unigene lengths is shown in Fig 3b, with sequence lengths mainly distributed between 300–2,000 nt. Sequences longer than 2,000 nt accounted for 23.3% of the total, and this distribution was consistent with the expected sequencing results.

**Table 2:** Statistics of Assembly Results.

Type	Total	≥500 bp	≥1000 bp	N50	Total length	Maximum length	Minimum length	Average length
Unigene	91627	62311	39712	2269	12381122	16933	301	1351.25

The assembled Unigenes were annotated in functional databases such as NR, KOG, GO, Swiss-Prot, eggNOG, KEGG, and Pfam. The results are shown in Fig 3c, with the largest number of Unigenes annotated in the NR database, followed by eggNOG, Swiss-Prot, GO, Pfam, KOG, and KEGG. A total of 45,772 Unigenes (49.95%) were annotated in the NR database (Fig 3d), with the highest proportion of Unigenes annotated to the homologous species Nyssa (also known as Nyssa sylvatica), accounting for 17.75%, followed by Cynara cardunculus, accounting for 9.32%. In the KEGG database, 10,029 Unigenes (10.95%) were annotated (Fig 3e), mainly divided into six categories, with the largest number of genes annotated to metabolism, totaling 7,176. Within this category, carbohydrate metabolism (1,810), amino acid metabolism (968), lipid metabolism (958), and energy metabolism (864) accounted for relatively high proportions. In the KOG database, 26,752 genes (29.20%) showed significant matches (Fig 3f). In the GO database, a total of 29,903 genes (32.64%) were matched (Fig 3g), and the genes were classified into three major categories: cellular components (26,478), biological processes (24,319), and molecular functions (25,709). In the eggNOG database, 42,305 Unigenes (46.17%) were annotated, and eggNOG categorized them into 26 specific groups, with the largest number of genes belonging to post-translational modification, protein turnover, and chaperones (2,931), followed by signal transduction mechanisms (2,357), transcription (2,192), replication, recombination, and repair (1,901), carbohydrate transport and metabolism (1,834), and translation, ribosomal structure, and biogenesis (1,665). Significant matches were found for 34,154 (37.28%) and 28,723 (31.35%) genes in the Swiss-Prot and Pfam databases, respectively.

### Analysis of unigene expression levels

**Fig 4:**
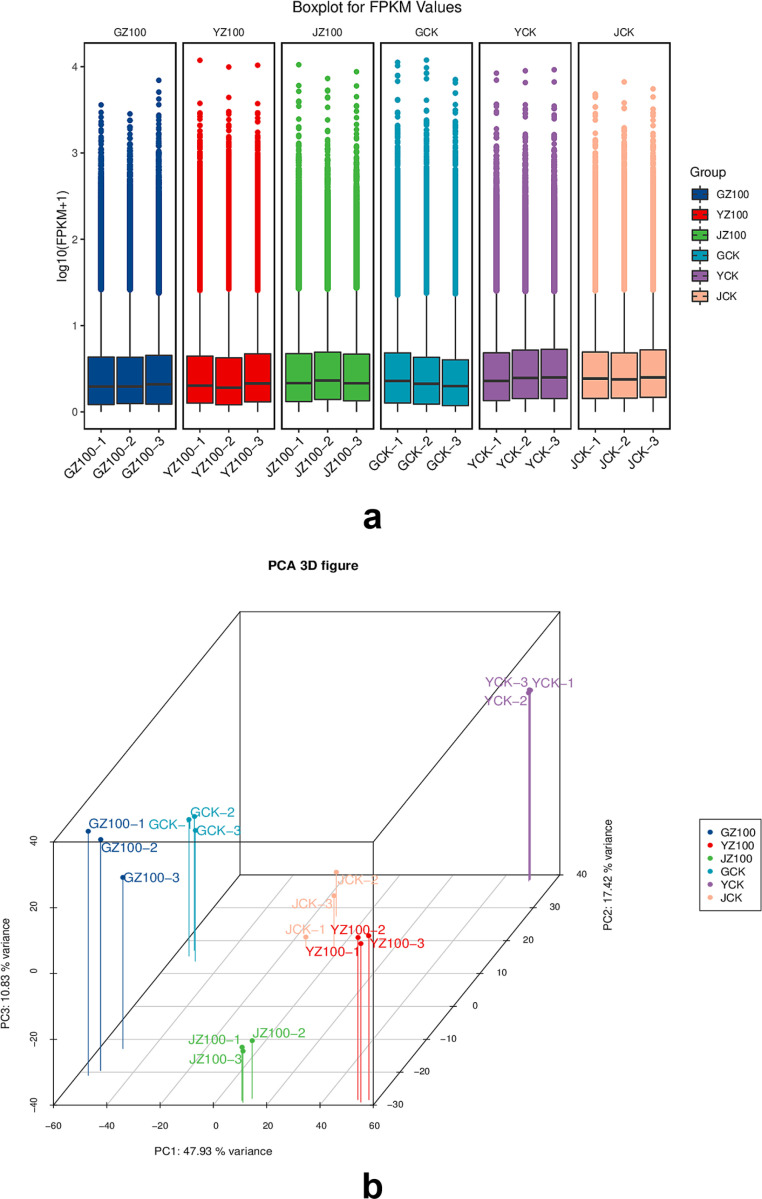
Analysis of Unigene Expression Levels and Principal Component Analysis. (a) Boxplot of FPKM Values; The boxplot in each region represents five statistical quantities (from top to bottom: maximum, third quartile, median, first quartile, and minimum); (b) PCA (Principal Component Analysis) Plot.

Fig 4a shows the boxplot of FPKM values for genes in each sample. From Fig 4a, it can be concluded that the FPKM values of genes in the six groups of samples were generally consistent in the boxplot, indicating that the expression levels of these genes were similar across samples and were less affected by technical and biological variations. This consistency supports their use in subsequent analysis. Principal component analysis of the samples (Fig 4b) demonstrates that the *R. palustris* group can be clearly separated from the control group, indicating that the biological replicates of *R. palustris* treatment were reliable and that the differences between groups were significant.

### Analysis of DEGs in different tissues of *C. pilosula*

**Fig 5:**
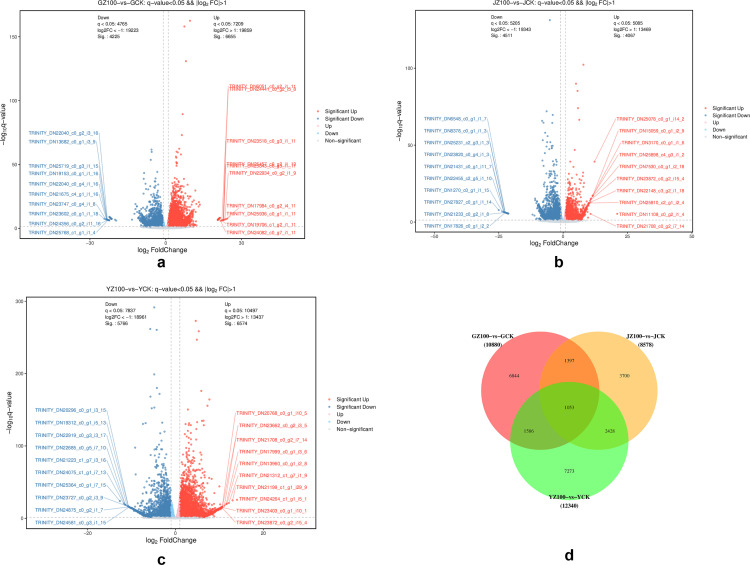
Volcano map and Venn diagram of differentially expressed genes in the roots, stems, and leaves of *Codonopsis pilosula.* Figs a-c represent the volcano plots of differential genes in the roots, stems, and leaves, respectively. In these plots, gray dots indicate non-differentially expressed Unigenes, red dots represent significantly upregulated differential Unigenes, and blue dots represent significantly downregulated differential Unigenes. Fig d shows the common, specific, and differential genes across the different comparison groups.

According to the screening criteria for the DEGs, a fold change factor of ≥2 and a q-value of <0.05 were used. A total of 10,880, 8,578, and 12,340 DEGs were identified through transcriptome sequencing of the root, stem, and leaf tissues of *C. pilosula* in response to *R. palustris* treatment, respectively. The highest number of DEGs was observed in the leaves (Fig 5a), with 6,574 DEGs upregulated and 5,766 DEGs downregulated. The root had the second highest number of DEGs (Fig 5b), with 6,655 DEGs upregulated and 4,225 DEGs downregulated. The lowest number of DEGs was found in the stems (Fig 5c), with 4,067 DEGs upregulated and 4,511 DEGs downregulated. Further analysis revealed 1,053 common DEGs among the three groups (Fig 5d). These results suggest that the application of *R. palustris* caused significant differences in the transcriptional profiles of gene expression across the various tissues of *C. pilosula*, indicating a tissue-specific response.

### GO functional enrichment analysis of DEGs

**Fig 6:**
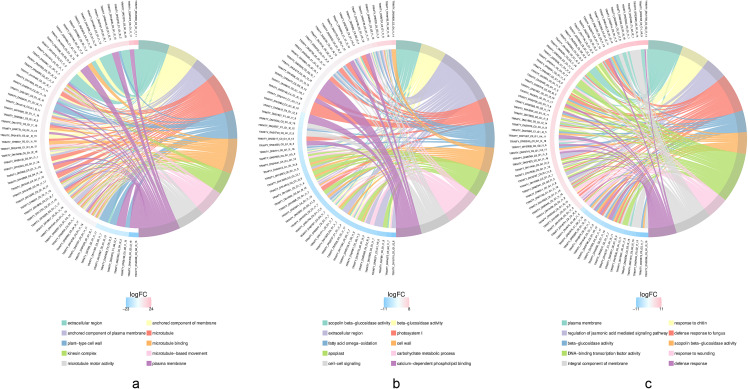
Top 10 Gene Ontology Enrichment Chord Diagram. Figs a-c represent the top 10 GO terms enriched by DEGs in the roots, stems, and leaves of *C. pilosula,* respectively. The left side shows the 10 genes with the largest |logFC| values in each category, while the right side reflects the composition of the categories. The middle lines indicate the correspondence between categories and genes. The outer heatmap represents the logFC values of the corresponding genes.

To further explore the potential gene functions of DEGs in different tissues treated with *R. palustris*, this study conducted a Gene Ontology (GO) functional enrichment analysis of these DEGs. In the stems, leaves, and roots of *C. pilosula*, 3,969, 5,199, and 5,272 DEGs, respectively, were enriched into three categories of GO terms. The top 10 GO terms with the smallest q-values in each differentially grouped term were selected (Fig 6), and these genes were highly similar in function to the gene annotations in the roots. The DEGs in the roots were mainly enriched in molecular function categories such as beta-glucosidase activity, UDP-glycosyltransferase activity, oxidoreductase activity, and beta-fructofuranosidase activity. They were annotated to cellular components such as the integral component of the membrane, plasma membrane, photosystem I, cell wall, and plant-type cell wall. They were also annotated to biological processes such as defense response, auxin-activated signaling pathway, carbohydrate metabolic processes, photosynthesis, light harvesting, and maltose catabolic processes. Notably, a larger number of DEGs related to sugar metabolism and photosynthesis were enriched in the roots and leaves, suggesting that after *R. palustris* treatment, entries related to sugar metabolism and photosynthesis may play a crucial role in regulating the accumulation of active ingredients in the roots and leaves of *C. pilosula*.

### KEGG pathway enrichment analysis of DEGs

**Fig 7:**
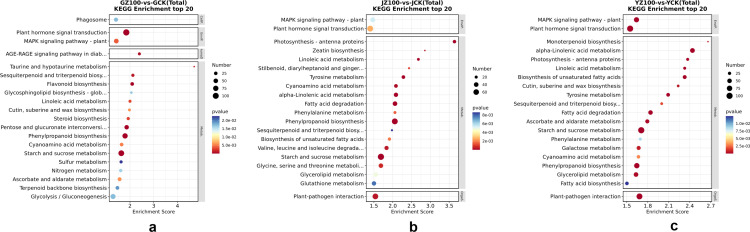
Top 20 Bubble Chart of Kyoto Encyclopedia of Genes and Genomes Enrichment. Figs a-c represent the bubble charts of the top 20 enriched differential genes in the roots, stems, and leaves of *Codonopsis pilosula*, respectively. The horizontal axis, labeled “Enrichment Score,” represents the enrichment score. Larger bubbles indicate a higher number of differentially expressed protein-coding genes included in the entry. The bubble color changes from blue to white, yellow, and red, with smaller p-values (indicating higher significance) represented by warmer colors.

To further uncover the mechanism of CPPS accumulation induced by *R. palustris* application, KEGG pathway enrichment analysis was performed on the screened DEGs. Pathway analysis was conducted on the differentially expressed protein-coding genes using the KEGG database, in conjunction with KEGG annotation results. A total of 1,522 DEGs were annotated in the KEGG Pathway database, involving 125 metabolic pathways. Among the top 20 metabolic pathways (Fig 7), those with significant enrichment of DEGs in the roots, stems, and leaves of *C. pilosula* included plant hormone signal transduction, starch and sucrose metabolism, phenylpropanoid biosynthesis (related to flavonoids), MAPK signaling pathway (plant), pentose and glucuronate interconversions, plant-pathogen interaction, glycolysis/gluconeogenesis, and others. It is hypothesized that these metabolic pathways may respond to the involvement of *R. palustris* in improving the agronomic traits of *C. pilosula* and accumulating the active components of CPPS.

### DEGs enriched in starch and sucrose metabolic pathways

**Fig 8:**
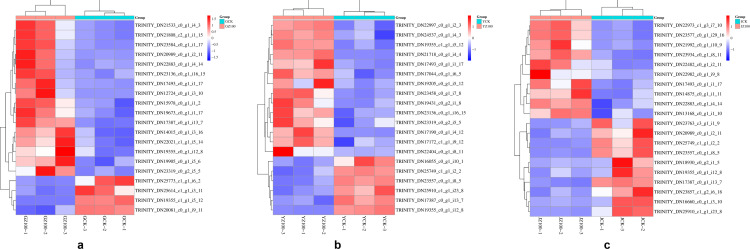
Clustering heatmap of FPKM values of differentially expressed genes involved in starch and sucrose metabolism pathways. Figs a-c represent the FPKM values of differentially expressed genes in the starch and sucrose metabolism pathways in the roots, leaves, and stems of *Codonopsis pilosula*, respectively. The horizontal axis represents samples, and the vertical axis represents genes, with red indicating up-regulation and blue indicating down-regulation.

When *R. palustris* diluted 100 times was used for root irrigation and spraying on *C. pilosula*, the DEGs in its roots, stems, and leaves were significantly enriched in starch and sucrose metabolic pathways. Consequently, KEGG map analysis of this metabolic pathway in the roots revealed that the enzymes of up-regulated genes in this pathway included fructokinase (scrK), hexokinase (HK), endoglucanase-1,3-β-D-glucosidase (EGLC), glucose-6-phosphate isomerase (GPI), β-fructofuranosidase (sacA), sucrose synthase (SUS), and glucose-1-phosphate adenylyltransferase (glgc). The enzymes of down-regulated genes included glucose phosphate mutase (pgm), uridine diphosphate glucose pyrophosphorylase (ugp), sucrose phosphate synthase, and trehalose-6-phosphate synthase (otsB). Based on the analysis of FPKM values of the genes encoding enzymes in this pathway in the roots (Fig 8a), it was found that the genes (TRINITY_DN17387_c0_g1_i13_7, TRINITY_DN19905_c0_g1_i5_6) encoding SUS were both up-regulated and highly expressed compared with the distilled water control group; the gene (TRINITY_DN14015_c0_g1_i3_16) encoding HK and the genes (TRINITY_DN22021_c1_g1_i5_14, TRINITY_DN23319_c0_g2_i5_5) encoding sacA were also up-regulated and highly expressed compared with the distilled water control group. In stems, the enzymes of up-regulated genes included scrK, sucrose-phosphate synthase, glgce, endoglucanase-1,3-β-D-glucosidase, and β-glucosidase, while the enzymes of down-regulated genes included sacA, SUS, pgm, and GPI. According to the FPKM values of the genes encoding enzymes in this pathway (Fig 8b), it was found that the genes (TRINITY_DN17493_c0_g1_i1_17, TRINITY_DN22883_c0_g1_i4_14) encoding scrK were both up-regulated and highly expressed compared with the distilled water control group. In leaves, the enzymes of up-regulated genes were scrK, otsB, α,α-trehalase, sacA, and sucrose phosphate synthetase. The enzymes of down-regulated genes included pgm, SUS, EGLC, and 1,4-α-glucan branchase, which were analyzed according to the FPKM values of the genes encoding these enzymes in this pathway (Fig 8c). It was observed that the genes (TRINITY_DN17044_c0_g1_i6_5, TRINITY_DN17493_c0_g1_i1_17) encoding scrK were up-regulated and highly expressed compared with the distilled water control group. The genes (TRINITY_DN19355_c1_g1_i5_12, TRINITY_DN22997_c0_g1_i2_3) encoding sacA were also up-regulated and highly expressed compared to the distilled water control group. Notably, the gene (TRINITY_DN17387_c0_g1_i13_7) encoding SUS and the gene (TRINITY_DN21503_c0_g1_i7_12) encoding glyceraldehyde-3-phosphate dehydrogenase (NADPH) in the glycolysis/gluconeogenesis metabolic pathway were both significantly up-regulated and highly expressed in the roots, whereas they were down-regulated in the stems and leaves. This result was consistent with the observation that the content of the active ingredient CPPS in the roots of *C. pilosula* was higher than in the stems and leaves. The above results indicate that the exogenous induction of *R. palustris* activated key enzymes (HK, SUS, scrK, sacA, GPI, Gapdh) in the biosynthetic pathway of *C. pilosula*, up-regulating the expression of genes that control these enzymes and promoting the accumulation of CPPS, the active ingredient of *C. pilosula*.

### DEGs enriched in plant hormone signal transduction pathways

**Fig 9:**
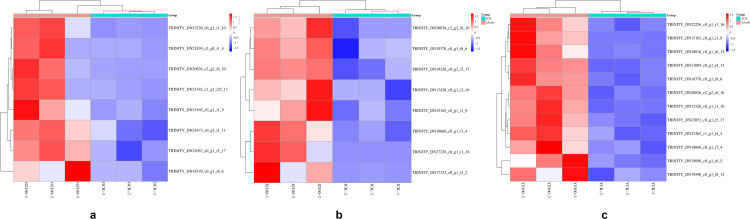
A cluster heatmap of FPKM values for differentially expressed genes involved in the jasmonic acid signal transduction pathway. (a), (b), and (c) respectively display the FPKM values of differentially expressed genes in the roots, stems, and leaves of *Codonopsis pilosula* within this pathway. In the heatmap, red represents up-regulation, while blue represents down-regulation.

Plant hormones, as signaling molecules, play a key role in mediating plant defense responses to both biotic and abiotic stresses. Through KEGG enrichment pathway analysis, following the root irrigation and spraying of *R. palustris*, the DEGs in the plant hormone signal transduction pathway were primarily enriched in roots, followed by leaves and stems. Specifically, in the roots, 110 DEGs were significantly enriched in the plant hormone signal transduction pathway. Of these, 38 DEGs were significantly enriched in the auxin signaling pathway, mainly encoding auxin-responsive protein IAA and auxin-responsive GH3 gene families (S1 Table). Additionally, 15 DEGs were enriched in the zeatin biosynthesis pathway, primarily encoding two-component response regulators of the ARR-A family and histidine-containing phosphotransfer proteins (S1 Table). In the leaves, 98 DEGs were significantly enriched in the plant hormone signal transduction pathway, with 32 DEGs belonging to the auxin signaling pathway. These primarily encoded auxin-responsive proteins IAA and members of the SAUR protein family (S2 Table). Furthermore, 12 DEGs were enriched in zeatin biosynthesis, mainly encoding histidine-containing phosphotransfer proteins and two-component response regulators of the ARR-A family (S2 Table). In the stems, 66 DEGs were enriched in the plant hormone signal transduction pathway. Of these, 25 DEGs belonged to the auxin signaling pathway, primarily encoding auxin-responsive proteins IAA and members of the SAUR protein family (S3 Table). Additionally, 6 DEGs were associated with the zeatin biosynthesis pathway, encoding histidine-containing phosphotransfer proteins and two-component response regulators of the ARR-A family (S3 Table). Moreover, this study found that differential genes enriched in the jasmonic acid signal transduction pathway were observed in roots, stems, and leaves, with all DEGs showing up-regulated expression. In the roots, the FPKM values of genes (TRINITY_DN22052_c0_g1_i5_17, TRINITY_DN23473_c0_g5_i1_11) encoding proteins containing the jasmonic acid ZIM domain (JAZ) and the coronatine-insensitive protein 1 (COI1) gene (TRINITY_DN21839_c3_g8_i1_4) were significantly up-regulated compared to the distilled water control group (Fig 9a). In the stems, the expression levels of genes (TRINITY_DN17426_c0_g1_i2_16, TRINITY_DN19320_c0_g2_i5_17, TRINITY_DN20836_c2_g2_i6_10) encoding proteins containing the jasmonic acid ZIM domain (JAZ) were also significantly up-regulated compared to the control group (Fig 9b). In the leaves, the expression levels of genes (TRINITY_DN18370_c0_g1_i8_6, TRINITY_DN20836_c2_g2_i6_10) encoding proteins containing the jasmonic acid ZIM domain (JAZ) were significantly up-regulated compared to the control group (Fig 9c). Furthermore, the FPKM values of genes (TRINITY_DN12520_c0_g1_i1_10, TRINITY_DN21004_c0_g1_i4_15) encoding the transcription factor MYC2 (MYC2) were also significantly up-regulated compared to the distilled water control group. These results suggest that *R. palustris* may enhance the agronomic traits of *C. pilosula* by promoting the expression of key enzyme and receptor genes in certain plant hormone signal transduction pathways, particularly the jasmonic acid signal transduction pathway. This could indirectly contribute to the increased accumulation of CPPS.

### qRT-PCR expression validation

**Fig 10:**
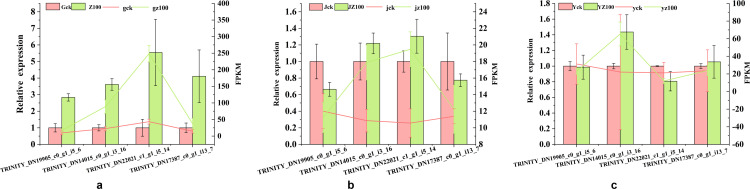
Relative expression levels of four Unigenes in the roots (a), stems (b), and leaves (c) of Codonopsis pilosula treated with Rhodopseudomonas palustris.

To thoroughly evaluate the accuracy and reproducibility of RNA-seq technology in the transcriptome study of *C. pilosula*, this study selected four key enzyme genes (TRINITY_DN19905_c0_g1_i5_6, TRINITY_DN14015_c0_g1_i3_16, TRINITY_DN22021_c1_g1_i5_14, TRINITY_DN17387_c0_g1_i13_7) closely related to the CPPS synthesis pathway from the database as targets. The purpose was to validate the results of the transcriptome data analysis using qRT-PCR (quantitative real-time reverse transcription polymerase chain reaction) technology (Fig 10). The results showed that the relative expression levels of the genes verified by qRT-PCR were consistent with the corresponding FPKM trends observed in the transcriptome database. Therefore, the transcriptome sequencing data obtained in this study on the regulation of *C. pilosula* in response to *R. palustris* are reliable.

## _Discussion_

As a microbial agent, *R. palustris* has unique ecological functions. It can not only optimize the soil micro-ecological environment, reduce the use of chemical fertilizers and pesticides, but also improve plant stress resistance, enhance immunity, and increase crop yields. In this study, the application of *R. palustris* during the planting of *C. pilosula* improved its agronomic traits. Changes in the agronomic traits of plants are the most direct indicators of growth and development, as they reflect the growth rate and adaptability of plants [38–40]. These results are similar to those reported by Jiayu Chang et al., who used *R. palustris* in planting *Astragalus membranaceus* and observed improved agronomic traits [18]. This suggests that the agronomic traits of *C. pilosula* can be enhanced by root irrigation and spraying with *R. palustris* (Fig 1) and that these results are reliable. Through the application of *R. palustris*, the diameter of the main root increased by 1.46 times compared to the distilled water control group, and the number of leaves increased by 0.9 times. It is noteworthy that, compared to the distilled water control group, the length of the root, leaf length, and plant height increased by 0.73, 0.81, and 0.45 times, respectively (Fig 1). Additionally, the content of CPPS in the roots, stems, and leaves of *C. pilosula* was measured, and the results showed that, compared to the distilled water control group, the polysaccharide content in the *R. palustris* treatment group increased by 90.22%, 61.11%, and 20.00%, respectively (Fig 2). This indicates that *R. palustris* induces significant changes in the agronomic traits of *C. pilosula* and influences the biosynthesis and accumulation of metabolites in various tissues. Consequently, it is necessary to quantify gene expression through transcriptome sequencing to identify key regulatory genes or important functional genes that impact the agronomic traits of *C. pilosula* and the accumulation of the active ingredient, CPPS.

In this study, *C. pilosula* was treated with *R. palustris* through root irrigation and spraying, followed by RNA-seq sequencing analysis of root, stem, and leaf tissues. This analysis yielded 10,880, 8,578, and 12,340 DEGs, respectively, in response to the application of *R. palustris*. Among these, 5,272, 3,916, and 5,119 DEGs in the roots, stems, and leaves were annotated in the GO database, respectively. The main enriched GO functions included carbohydrate metabolic processes, beta-glucosidase activity, UDP-glycosyltransferase activity, photosystem I, maltose catabolic processes, auxin-activated signaling pathways, and other functions. A total of 1,522, 1,202, and 1,616 DEGs in the roots, stems, and leaves were annotated in the KEGG database, respectively. KEGG pathway enrichment analysis of the selected DEGs revealed that, under the treatment of *R. palustris*, the DEGs of *C. pilosula* were significantly enriched in metabolic pathways such as plant hormone signal transduction, starch and sucrose metabolism, phenylpropanoid biosynthesis, flavonoid biosynthesis, MAPK signaling pathways, plant-pathogen interactions, and glycolysis/gluconeogenesis. Twelve DEGs involved in starch and sucrose metabolism and ten DEGs involved in plant hormone signal transduction (jasmonic acid signaling pathway) were identified, providing abundant data to support elucidating the molecular mechanism of *R. palustris* in regulating the accumulation of CPPS, the main active ingredient in *C. pilosula*.

Studies have shown that leaves are the primary site for photosynthesis in plants [41–43]. Sucrose, a crucial product of photosynthesis, is one of the essential substances involved in the biosynthesis of CPPS. It is distributed to different storage tissues through the action of various glycosyltransferases, leading to the accumulation of CPPS [44,45], This is consistent with the results of this study, After treatment with *R. palustris*, sucrose was synthesized in the leaves via enhanced photosynthesis and transported through the stems, where it was ultimately synthesized into CPPS under the action of multiple glycosyltransferases, accumulating in the roots. which show that the content of CPPS in the roots differs from that in the stems and leaves. KEGG analysis revealed that the tissue with the largest number of DEGs enriched in starch and sucrose metabolism was the leaves, followed by the roots, which is consistent with the functional study of leaf-derived organs by Wang R, et al. [46]. Based on this analysis, it was speculated that, after the root irrigation and spraying treatment with *R. palustris*, DEGs were significantly enriched in the starch and sucrose metabolic pathways. SUS and sacA catalyze the mutual conversion between UDP-Glu and sucrose [47,48], while scrK and HK catalyze the reversible conversion between D-fructose and D-fructose-6-phosphate [49]. The content of CPPS in the *R. palustris* group was higher than in the distilled water control group, which was also supported by a detailed analysis of the DEGs in the *R. palustris* group. As shown in Fig 7, a large number of DEGs in the *R. palustris* group were involved in starch and sucrose metabolism. The expression levels of these genes in the *R. palustris* group were higher than in the distilled water control group. This could explain the higher content of CPPS in the *R. palustris* group. For example, the genes encoding SUS (TRINITY_DN17387_c0_g1_i13_7, TRINITY_DN19905_c0_g1_i5_6), sacA (TRINITY_DN22021_c1_g1_i5_14, TRINITY_DN23319_c0_g2_i5_5), scrK (TRINITY_DN17493_c0_g1_i1_17), and HK (TRINITY_DN14015_c0_g1_i3_16) involved in starch and sucrose metabolic pathways showed higher expression levels in the roots (Fig 8a). Wang C, et al. conducted transcriptome sequencing on *Polygonatum cyrtonema* to analyze the mechanism of polysaccharide accumulation, and they found that the SUS gene was involved in polysaccharide biosynthesis [50]. Furthermore, this study identified the upregulation of genes such as HK, scrK, and sacA during the process of CPPS accumulation, which may explain why CPPS content in *R. palustris* was higher than in the distilled water control group. Additionally, compared to the stems and leaves of *C. pilosula*, the SUS gene (TRINITY_DN17387_c0_g1_i13_7) exhibited higher expression levels in the roots, consistent with the finding that CPPS content was higher in the roots than in the stems and leaves. Therefore, *R. palustris* enhances the content of important substances such as sucrose and UDP-sugar in the CPPS synthesis process by regulating the activities of enzymes such as SUS, sacA, scrK, and HK, which jointly promote the accumulation of CPPS through the action of glycosyltransferases.[1]

Recent studies have shown that plant hormones, as signaling molecules, play an important regulatory role in the process of plant growth and development [51–53], including auxin, jasmonic acid (JA), cytokinin, abscisic acid, and ethylene. Through KEGG pathway analysis, this study found that the DEGs were significantly enriched in plant hormone signal transduction pathways, with all DEGs in the *R. palustris* group upregulated in the JA signaling pathway (Fig 9). The JA signaling pathway plays a key role in mediating plant responses and defenses against biotic and abiotic stresses [54,55]. Additionally, the JA signaling pathway positively regulates the initiation and elongation of root hairs. First, the JA signaling pathway binds to receptor proteins (such as F-box protein COI1) to form a receptor complex. This complex interacts with JAZ proteins (jasmonate-ZIM domain), leading to the ubiquitination and degradation of JAZ proteins. JAZ proteins act as key inhibitors in the JA signaling pathway, and their degradation relieves the inhibition of downstream transcription factors (such as MYC2), thereby activating the expression of downstream genes [56–58]. This study found that, compared to the water control group, the genes encoding COI1 (TRINITY_DN21839_c3_g8_i1_4), JAZ (TRINITY_DN22052_c0_g1_i5_17), and MYC2 (TRINITY_DN12520_c0_g1_i1_10) in the JA synthesis pathway exhibited higher expression levels in roots, stems, and leaves in the *R. palustris* group. This may explain why the agronomic traits such as root length, root diameter, and leaf number in *C. pilosula* were superior to those in the distilled water control group. In studies on *Arabidopsis thaliana*, it was discovered that the JA signaling pathway plays a crucial role in plant development, regulating the expression of genes such as F-BOX (SCF) and COI1, while mediating responses to both biotic and abiotic stresses [59]. In research on melon roots, the activation of AP2/ERF, BBR/BPC, and HD-ZIP transcription factor family members in the JA signaling pathway was shown to promote the development of melon roots [60]. However, this study newly discovered that, in addition to regulating the COI1 gene, genes such as JAZ and MYC2 were upregulated in *R. palustris* groups. Therefore, the results of this study indicate that plant hormone signal transduction, particularly the JA signaling pathway, plays a crucial regulatory role in controlling the agronomic traits of *C. pilosula*. Under *R. palustris* treatment, JAZ, COI1, and MYC2 may positively regulate the JA signaling pathway in *C. pilosula*, thereby promoting increases in agronomic traits such as root length, root diameter, and leaf number, which indirectly enhances the accumulation of CPPS.

In addition, KEGG analysis showed that DEGs were significantly enriched in phenylpropanoid biosynthesis pathways, with the highest enrichment in the roots, followed by the leaves and stems. The phenylpropanoids of *C. pilosula* include Codonopsis glycosides I-IV, furanocoumarins, and syringin, which possess antitumor activity [61]. Codonopsis glycosides I-IV and syringin are sequentially synthesized by phenylalanine ammonia-lyase, 4-coumarate-CoA ligase (4CL), cinnamoyl-CoA reductase (CCR), cinnamyl alcohol dehydrogenase (CAD), catalase, and others [57]. According to the KEGG map, the FPKM values of genes encoding phenylalanine ammonia-lyase, 4CL, CCR, and CAD were upregulated compared to the distilled water control group, laying a foundation for further research on the accumulation mechanism of other active ingredients in *C. pilosula*. DEGs were also enriched in the MAPK signaling transduction pathway, which actively responds to environmental stress and regulates plant growth and development [62–64]. Therefore, we speculate that exogenous *R. palustris* induced and activated the expression of key enzyme genes in this pathway, which jointly promoted the accumulation of CPPS, the active ingredient of *C. pilosula*, and improved its agronomic traits.

## _Conclusion_

In order to further explore the effect of *R. palustris* on the agronomic traits of *C. pilosula* and the regulatory mechanism of key enzyme genes in the polysaccharide biosynthesis pathway, this study quantitatively analyzed the expression levels of key enzyme genes. The results showed that after treatment with *R. palustris*, the genes encoding HK, SUS, scrK, sacA, and GPI were significantly upregulated in the starch and sucrose metabolism pathways of *C. pilosula*, and their expression levels were high, whereas the genes encoding UGP2 and PGM showed no significant differences. Therefore, exogenous *R. palustris* activates the expression of key enzyme genes in certain metabolic pathways (starch and sucrose metabolism) of *C. pilosula*, thereby enhancing the accumulation of CPPS, the active ingredients of *C. pilosula*. In the plant hormone signal transduction, particularly the JA signaling pathway, the genes encoding JAZ, COI1, and MYC2 were found to have high expression levels. It is speculated that exogenous *R. palustris* induced the activation of plant hormone signal transduction, especially the expression of key enzyme genes in the JA signaling pathway, thereby improving the agronomic traits of *C. pilosula* and indirectly promoting the biosynthesis and accumulation of CPPS, the active component of *C. pilosula*. As a result, in the cultivation of *C. pilosula*, the application of *R. palustris* proved beneficial for improving the agronomic traits of *C. pilosula* and enhancing the accumulation of CPPS, its active ingredient. This treatment not only actively responded to environmental protection but also regulated key genes involved in the synthesis of pharmacological substances through transcriptional sequencing technology, laying the foundation for increasing the accumulation of CPPS, the effective ingredient of *C. pilosula*, through molecular means. With the increasing awareness of environmental protection, green ecological agriculture is becoming an important direction for the future development of agriculture. *R. palustris*, as a biofertilizer, not only enhances the growth efficiency of *C. pilosula* but also promotes soil health. The application of *R. palustris* helps restore and protect soil fertility, preventing soil degradation and pollution. Therefore, using *R. palustris* as an ecological agricultural management approach provides new insights and technical support for the sustainable development of the *C. pilosula* industry and the realization of ecological agriculture. In the future, breeding strategies for *C. pilosula* can be further optimized to ensure its good growth under a wider range of environmental conditions and to enhance the accumulation of its medicinal components. The combination of microbial inoculants and molecular biology techniques can precisely regulate agronomic traits and medicinal component production at the molecular level, thereby improving the quality and efficiency of the *C. pilosula* industry.

Although this study reveals the effects of *R. palustris* on the agronomic traits and polysaccharide synthesis of *C. pilosula* and identifies some key enzyme genes, the underlying mechanisms are not yet fully understood. It remains unclear how *R. palustris* specifically regulates these key genes and influences plant growth and metabolism. Future research will need to employ genetic engineering, gene editing, or molecular marker technologies to further explore the molecular interactions between the microorganisms and the plants and to clarify their mechanisms.

## Supporting information

S1 Table Expression patterns of differential genes in root plant hormone signal transduction. (PDF)

S2 Table Expression patterns of differential genes in leaf plant hormone signal transduction pathway. (PDF)

S3 Table Expression patterns of differential genes in stem plant hormone signal transduction pathway. (PDF)

S1 File All original data of this experiment. (ZIP)
