# Supplementary material for: The effects of Rhodopseudomonas palustris on the improvement of agronomic traits and key enzyme-coding genes related to polysaccharide biosynthesis in Codonopsis pilosula
Source: PLoS One. 2025 Jun 3;20(6):e0319989. doi: 10.1371/journal.pone.0319989 (PMC12132940; doi:10.1371/journal.pone.0319989)
Supplement: S1 Table — (PDF) [file pone.0319989.s001.pdf]

**S1 Table Expression patterns of differential genes in root plant hormone signal transduction pathway**

| Gene id | Encoding enzyme/protein | Up/Down |
|---|---|---|
| TRINITY_DN19611_c0_g1_i2_8、TRINITY_DN20839_c0_g1_i1_1 TRINITY_DN21863_c0_g4_i1_14、TRINITY_DN22844_c1_g1_i13_5 TRINITY_DN23762_c0_g1_i10_17、TRINITY_DN25570_c4_g1_i4_13 | Auxin influx carriers (AUX1 and LAX family) | Up |
| TRINITY_DN24278_c1_g6_i2_4、TRINITY_DN24669_c0_g3_i13_6 | Auxin influx carriers (AUX1 and LAX family) | Down |
| TRINITY_DN17299_c0_g2_i1_10 | Transport inhibitor response protein 1 | Up |
| TRINITY_DN13518_c0_g1_i2_5、TRINITY_DN16102_c0_g3_i2_8 TRINITY_DN16672_c0_g1_i2_18、TRINITY_DN17201_c0_g2_i5_18 TRINITY_DN18603_c0_g1_i2_11、TRINITY_DN19586_c2_g1_i1_18 TRINITY_DN20866_c0_g2_i1_8、TRINITY_DN22023_c0_g1_i1_4 TRINITY_DN22196_c5_g1_i2_13 | Auxin-responsive protein IAA | Up |
| TRINITY_DN15610_c0_g1_i1_4、TRINITY_DN21227_c1_g1_i9_11 TRINITY_DN24187_c1_g4_i1_7 | Auxin-responsive protein IAA | Down |
| TRINITY_DN16452_c0_g1_i3_14、TRINITY_DN18812_c0_g1_i9_17 | Auxin response factors | Up |
| TRINITY_DN22806_c0_g1_i9_17、TRINITY_DN25700_c0_g2_i9_4 | Auxin response factors | Down |
| TRINITY_DN11717_c0_g1_i2_10、TRINITY_DN12027_c0_g1_i2_1 TRINITY_DN12813_c0_g1_i1_1、TRINITY_DN13083_c0_g1_i2_9 TRINITY_DN23225_c1_g3_i7_9、TRINITY_DN25233_c1_g1_i3_15 TRINITY_DN8188_c0_g1_i1_16 | Auxin-responsive GH3 gene family | Up |
| TRINITY_DN10124_c0_g2_i1_10、TRINITY_DN2296_c0_g1_i1_9 TRINITY_DN23859_c1_g7_i1_6 | SAUR family proteins | Up |
| TRINITY_DN12581_c0_g1_i1_9、TRINITY_DN21370_c0_g2_i1_4 TRINITY_DN4951_c0_g1_i1_9 | SAUR family proteins | Down |
| TRINITY_DN19221_c0_g1_i3_13、TRINITY_DN22239_c0_g1_i2_15 | Arabidopsis histidine kinase | Down |
| TRINITY_DN22080_c0_g1_i1_7、TRINITY_DN23260_c0_g1_i7_4 | Arabidopsis histidine kinase | Up |
| TRINITY_DN16141_c0_g2_i4_16、TRINITY_DN16877_c0_g1_i7_7 TRINITY_DN17074_c0_g1_i5_15、TRINITY_DN19446_c0_g1_i7_13 | Histidine-containing phosphotransfer proteins | Up |
| TRINITY_DN21590_c0_g1_i7_4、TRINITY_DN22968_c0_g1_i1_8 | Histidine-containing phosphotransfer proteins | Down |
| TRINITY_DN25264_c0_g3_i3_7 | Two-component response regulator ARR-B family | Up |
| TRINITY_DN13667_c0_g1_i1_14、TRINITY_DN21950_c0_g1_i2_18 TRINITY_DN22894_c0_g1_i4_5、TRINITY_DN23146_c2_g2_i7_1 | Two-component response regulator ARR-B family | Up |
