# Supplementary material for: The effects of Rhodopseudomonas palustris on the improvement of agronomic traits and key enzyme-coding genes related to polysaccharide biosynthesis in Codonopsis pilosula
Source: PLoS One. 2025 Jun 3;20(6):e0319989. doi: 10.1371/journal.pone.0319989 (PMC12132940; doi:10.1371/journal.pone.0319989)
Supplement: S2 Table — (PDF) [file pone.0319989.s002.pdf]

S2 Table Expression patterns of differential genes in leaf plant hormone signal transduction pathway

Gene id	Encoding enzyme/protein	Up/Down
TRINITY_DN23439_c1_g2_i13_1、TRINITY_DN24669_c0_g4_i2_6 TRINITY_DN25570_c4_g1_i4_13	Auxin influx carriers (AUX1 and LAX family)	Up
TRINITY_DN24278_c1_g6_i2_4、TRINITY_DN24669_c0_g3_i13_6	Auxin influx carriers (AUX1 and LAX family)	Down
TRINITY_DN11746_c0_g1_i1_17、TRINITY_DN13042_c0_g1_i1_13 TRINITY_DN16510_c0_g1_i5_8、TRINITY_DN19742_c0_g1_i12_15 TRINITY_DN19846_c0_g1_i2_12、TRINITY_DN21053_c1_g1_i1_17 TRINITY_DN21227_c1_g1_i9_11、TRINITY_DN21654_c2_g1_i2_14	Auxin-responsive protein IAA	Up
TRINITY_DN24334_c0_g1_i3_3、TRINITY_DN25206_c0_g1_i6_4	Auxin response factors	Up
TRINITY_DN22806_c0_g1_i9_17、TRINITY_DN24110_c0_g2_i17_14	Auxin response factors	Down
TRINITY_DN13083_c0_g1_i2_9、TRINITY_DN8188_c0_g1_i1_16	Auxin-responsive GH3 gene family	Down
TRINITY_DN23225_c1_g3_i7_9、TRINITY_DN23813_c0_g3_i1_13 TRINITY_DN25233_c1_g1_i3_15	Auxin-responsive GH3 gene family	Up
TRINITY_DN10124_c0_g1_i1_10、TRINITY_DN10124_c0_g2_i1_10 TRINITY_DN12204_c0_g1_i1_5、TRINITY_DN21370_c0_g2_i1_4 TRINITY_DN2296_c0_g1_i1_9、TRINITY_DN26153_c0_g1_i1_18 TRINITY_DN30050_c0_g1_i1_14、TRINITY_DN6702_c0_g1_i1_4 TRINITY_DN9626_c0_g1_i1_4、TRINITY_DN9886_c0_g1_i1_12	SAUR family proteins	Up
TRINITY_DN23260_c0_g1_i7_4	Arabidopsis histidine kinase	Down
TRINITY_DN24355_c1_g1_i10_15	Histidine-containing phosphotransfer proteins	Down
TRINITY_DN16141_c0_g2_i4_16、TRINITY_DN17074_c0_g1_i5_15 TRINITY_DN19173_c0_g4_i1_16、TRINITY_DN19861_c0_g1_i2_4 TRINITY_DN23962_c0_g2_i1_1	Histidine-containing phosphotransfer proteins	Up
TRINITY_DN20842_c1_g1_i11_9	Two-component response regulator ARR-B family	Down
TRINITY_DN21647_c1_g2_i6_10、TRINITY_DN21782_c0_g1_i2_11 TRINITY_DN22083_c0_g1_i2_16、TRINITY_DN23146_c2_g2_i7_1	Two-component response regulator ARR-B family	Up
