# Supplementary material for: The effects of Rhodopseudomonas palustris on the improvement of agronomic traits and key enzyme-coding genes related to polysaccharide biosynthesis in Codonopsis pilosula
Source: PLoS One. 2025 Jun 3;20(6):e0319989. doi: 10.1371/journal.pone.0319989 (PMC12132940; doi:10.1371/journal.pone.0319989)
Supplement: S3 Table — (PDF) [file pone.0319989.s003.pdf]

S3 Table Expression patterns of differential genes in stem plant hormone signal transduction pathway

Gene id	Encoding enzyme/protein	Up/Down
TRINITY_DN23762_c0_g1_i10_17	Auxin influx carriers (AUX1 and LAX family)	Down
TRINITY_DN15610_c0_g1_i1_4、TRINITY_DN18603_c0_g1_i2_11<br>TRINITY_DN19586_c2_g1_i1_18	Auxin-responsive protein IAA	Up
TRINITY_DN20820_c2_g4_i2_5、TRINITY_DN22941_c1_g1_i3_7<br>TRINITY_DN24187_c1_g4_i1_7	Auxin-responsive protein IAA	Down
TRINITY_DN22806_c0_g1_i9_17、TRINITY_DN24110_c0_g2_i17_14<br>TRINITY_DN25700_c0_g2_i9_4	Auxin response factors	Down
TRINITY_DN11717_c0_g1_i2_10、TRINITY_DN12027_c0_g1_i2_1<br>TRINITY_DN23225_c1_g3_i7_9、TRINITY_DN25233_c1_g1_i3_15	Auxin-responsive GH3 gene family	Up
TRINITY_DN10124_c0_g1_i1_10、TRINITY_DN12204_c0_g1_i1_5<br>TRINITY_DN21292_c2_g1_i2_10、TRINITY_DN22587_c0_g1_i2_3<br>TRINITY_DN22830_c1_g1_i1_17、TRINITY_DN23433_c2_g2_i2_9<br>TRINITY_DN24038_c0_g2_i1_6、TRINITY_DN26153_c0_g1_i1_18	SAUR family proteins	Up
TRINITY_DN2275_c0_g1_i1_7、TRINITY_DN29826_c0_g1_i1_6<br>TRINITY_DN32205_c0_g1_i1_7	SAUR family proteins	Down
TRINITY_DN16141_c0_g2_i4_16、TRINITY_DN19173_c0_g4_i1_16	Histidine-containing phosphotransfer proteins	Up
TRINITY_DN22705_c0_g1_i3_5	Two-component response regulator ARR-B family	Down
TRINITY_DN13667_c0_g1_i1_14、TRINITY_DN21782_c0_g1_i2_11<br>TRINITY_DN22083_c0_g1_i2_16	Two-component response regulator ARR-B family	Up
